# A Sustainable Approach: Repurposing Red Beetroot Peels for Innovative Meringue Products

**DOI:** 10.3390/foods14020317

**Published:** 2025-01-18

**Authors:** Oana Emilia Constantin, Florina Stoica, Silvia Lazăr (Mistrianu), Doina Georgeta Andronoiu, Mihaela Turturică, Nicoleta Stănciuc, Roxana Nicoleta Rațu, Constantin Croitoru, Gabriela Râpeanu

**Affiliations:** 1 Integrated Center for Research, Expertise and Technological Transfer in Food Industry, Faculty of Food Science and Engineering, Dunarea de Jos University of Galati, 111 Domnească Street, 800201 Galati, Romania; emilia.constantin@ugal.ro (O.E.C.); silvia.mistrianu@ugal.ro (S.L.); georgeta.andronoiu@ugal.ro (D.G.A.); mihaela.turturica@ugal.ro (M.T.); nicoleta.stanciuc@ugal.ro (N.S.); roxana.ratu@iuls.ro (R.N.R.); constantin.croitoru@asas.ro (C.C.); 2 Department of Pedotechnics, Faculty of Agriculture, “Ion Ionescu de La Brad” Iasi University of Life Sciences, 3 Mihail Sadoveanu Alley, 700489 Iasi, Romania; florina.stoica@iuls.ro; 3 Department of Food Technologies, Faculty of Agriculture, “Ion Ionescu de La Brad” Iasi University of Life Sciences, 3 Mihail Sadoveanu Alley, 700489 Iasi, Romania; 4 Academy of Agricultural and Forestry Sciences, 61 Marasti Blvd, 011464 Bucharest, Romania

**Keywords:** beetroot by-products, polyphenols, betalains, antioxidant activities, meringues, color, texture

## Abstract

With the increasing global demand for sustainable and eco-friendly food items, it is imperative to investigate alternate sources of natural pigments. The red beetroot (*Beta vulgaris* L.) is a traditional food in many countries and a rich bioactive compound known for its beneficial properties. Beetroot peel, a by-product of beetroot food processing, is often discarded, contributing to environmental damage. This research explores the potential of beetroot peel (BP) powder as a natural pigment in food products and its functional benefits. The study focuses on incorporating BP powder into meringues, aiming to create a value-added product with enhanced properties, particularly antioxidant activity. Various amounts of BP powder (4–10%) were added to meringue formulations, and the effects on the resulting meringues’ physicochemical properties, sensory qualities, and phytochemical profiles were assessed during 21 days of storage. The research revealed that BP powder, besides its function as a natural colorant and the pleasing pink hue it imparts to meringues, also enhances antioxidant activity due to its high phenolic concentration. BP powder was also incorporated to enhance the meringues’ overall sensory characteristics, improving their flavor and texture. The research findings indicate that BP has the potential to be used as a natural food ingredient to promote human health, resource-use efficiency, and a circular economy.

## 1. Introduction

The rapid growth of the human population in recent decades has led to a surge in food processing activities, generating significant agro-industrial by-products. Globally, the agro-food industry produces over 190 million tons of by-products annually [1]. One of the objectives of the United Nations for 2030 is to diminish food waste generation to attain a more sustainable global environment. Due to the growing concern and knowledge of this issue, scientists have been studying and proposing strategies to valorize these by-products by recovering substances and compounds from these matrices using various extraction procedures [2]. The valorization of beetroot peel constitutes a novel approach to mitigate agro-industrial waste in accordance with sustainability standards. By recycling these by-products, industries can minimize the environmental impact of waste disposal while concurrently recovering valuable substances such as betalains and polyphenols. This method reduces landfill utilization and greenhouse gas emissions while fostering the circular economy by converting by-products into valuable food product components, thereby offering a cost-efficient substitute for synthetic additions [3,4]. By-products are full of bioactive substances that can be used in the food, pharmaceutical, and cosmetic industries. Antioxidants, fibers, minerals, carotenoids, and other substances are included in this group [5].

Red beet (*Beta vulgaris* L.) is one of the most significant and nutrient-rich vegetables. This biennial plant is a member of the *Amaranthaceae-Chenopodiaceae* family and is believed to have originated in Asia and Europe [6]. Beets are cultivated worldwide. An estimated 275.49 million tons of beetroot were produced globally in 2018. Major beetroot producers include France, the United States, Russia, Germany, and Ukraine [7]. *Beta vulgaris (*beetroot or red beet) is rich in nutrients such as vitamins (A, C, E, K, and B), minerals (potassium, zinc, sodium, phosphorus, calcium, and magnesium), folic acid, and antioxidants like phenolic acids, flavonoids, carotenoids, betalains, and amino acids [3,5]. Several researchers stated that beetroot is a significant source of phytochemicals that promote health [8,9]. In addition to their anti-diabetic, cardio-vascular disease-lowering, hypertensive, and wound healing benefits, beetroot’s polyphenols, carotenoids, and vitamins possess antioxidant, anti-inflammatory, anticarcinogenic, and hepatoprotective properties [10].

Betalains, nitrogen-containing pigments, give beetroot its red color and serve as bioactive phytochemicals with reducing potential due to a cyclic amine-reactive group [11]. Betalains offer distinct benefits compared to other natural pigments, such as higher antioxidant activity, low toxicity, cost-effectiveness, health benefits, unique color spectrum, and pH tolerance. Betalains, primarily sourced from beets and a few other plants, face limitations such as heat sensitivity, light-induced degradation, and restricted availability. Known as “red beetroot” in the food industry, these pigments are used as natural colorants (E162) in products like tomato paste, jams, soups, cereals, and sweets [12].

Meringue is a popular confectionery product prepared by combining egg whites and sugar. Meringue is a Swiss, French, Polish, and Italian dessert. Due to the component ingredients, the main nutrients in meringue are protein and carbohydrates. It does not have a stable structure, so it has a short shelf life of about two weeks if stored properly. Meringue is a hygroscopic food that quickly absorbs water from the air. This is the basis for many desserts: soufflés, macarons, tiramisu, and mousse [13,14]. The color of this product is a significant quality element that consumers value. During its production, synthetic dyes such as Sunset Yellow, Ponceau 4R, and Tartrazine are used to enhance its appeal to consumers [15]. Anthocyanins are commonly found and widely utilized natural pigments that give a red-purple color. However, betalains are more resistant to high temperatures and have a wide range of pH stability. This makes them particularly appropriate for application in low-acid foods [16]. Multiple studies have shown the possibility of utilizing betalains as a substitute for synthetic colors, which might potentially have detrimental impacts on health [15,16]. Among the applications of betalains in culinary products are burgers, desserts, ice cream, preserves, jellies, soups, sauces, candies, beverages, and dairy products [17]. Water-soluble secondary metabolites, a class of betalamic acid derivatives, are composed of two subclasses: betacyanins (reddish-violet substances with a predominance of betanin 75–95%) and betaxanthins (yellow-orange substances with a predominance of violaxanthin I 95%) [18]. The appeal of betalains is derived from their capacity to color and the beneficial health effects that result from their potent antioxidant properties. Betalains are essential for preventing cancer and ailments associated with oxidative stress, as they possess substantial antioxidant activity [19]. In addition, betalains demonstrate anti-lipidemic effects [20] and significant antibacterial activity [21].

Research highlights the nutritional, color, and flavor benefits of red beetroot betalains, sparking interest in natural pigments due to the health risks of synthetic red colorants [10,22]. Beetroot processing generates significant waste, including peels, seeds, stems, and pomace, with the FAO estimating up to 1.3 billion tons of residues annually, amounting to one-third of total food production [23].

Manufacturers had previously disposed of these wastes or utilized them as animal feed or fertilizers. However, new research has revealed that they possess valuable bioactive components that can be used as food additives or in producing innovative functional food. Therefore, using beetroots and their by-products as a natural ingredient in various food products positively impacts human health and opens up the possibility of developing multiple enhanced product formulations. The economic significance of utilizing this by-product is currently a matter of concern. In light of the contemporary implementation of sustainability principles, there is a growing trend of developing novel high-value compounds derived from agro-food by-products. These compounds are then utilized in the production of food ingredients that possess the ability to preserve resources. As a result, this approach contributes to promoting resource-use efficiency and establishing a circular economy [24]. The peel of the beetroot contains a more significant amount of betanin and phenolic chemicals than the flesh and crown, suggesting the potential for reusing the peel. The peel of red beetroot is recognized for its elevated concentration of ferulic acid, a compound commonly seen in several species that contain betalain [25].

Recently, Kim et al. [26] fortified meringue cookies with tomato powder (5, 10, and 15%) and found that it improved the overall quality of the final product. Furthermore, other authors [27,28] enhanced the meringue cookies with red-fleshed pitaya peels and the flowers of *Amaranthus caudatus* and coffee exocarp, evidencing the potential as natural pigments of agri-food by-products in place of synthetic dyes in the manufacture of French meringue. Conversely, using beetroot by-products, less research has been conducted to improve meringue’s overall nutritional, phytochemical, textural, and color properties.

This study aimed to examine the feasibility of using BP powder as a natural source of bioactive compounds in meringues to enhance nutritional value and visual appearance. The current study aimed to develop value-added meringues by including freeze-dried beetroot peel (BP) powders of different proportions (4–10%) as a source of natural colorants and bioactive components. The effects of BP powder supplementation on the phytochemical and physicochemical content, sensory qualities, color, and textural features of the meringues were also examined. Therefore, the study demonstrates the potential for BP powder to replace synthetic colorants with natural pigments while enhancing meringues’ nutritional and sensory properties. Additionally, the valorization of beetroot by-products aligns with sustainability goals, promoting resource efficiency and a circular economy.

## 2. Materials and Methods

### 2.1. Reagents and Chemicals

Red Beets (*Beta vulgaris* ssp. *vulgaris* var. *conditiva* Rubiniu) were bought from a local farmer (2023 cultivation year) in Galați, Romania (Latitude: 45.43° N; Longitude: 28.03° E).

Powdered sugar and egg powder were purchased from a supermarket in Galați, Romania. Folin–Ciocalteu reagent, glacial acetic acid, HPLC purity methanol, 2,2-diphenyl-1-picrylhydrazyl (DPPH), ethanol, 6-hydroxy2,5,7,8 tetramethylchromane-2-carboxylic acid (Trolox), gallic acid, sodium hydroxide, aluminum chloride, and sodium carbonate were obtained from Sigma Aldrich Steinheim (Darmstadt, Germany). All the reagents used in the experiments were of analytical grade.

### 2.2. Beetroot Peel (BP) Powder Preparation and Its Chemical Composition

Beetroots were collected, cleaned with distilled water, and dried. The samples were hand-peeled, and the collected peels were cut into small pieces and preserved at −25 °C until lyophilization. The collected peels were lyophilized for 48 h at −42 °C up to a relative humidity of 8 ± 0.5% with a pressure of 0.10 mBar using Alpha 1–4 LD plus equipment (Christ, Germany). The freeze-dried peels were pulverized into a fine powder by sieving them through a 450 µm porosity screen using an MC 12 grinder from Producer Stephan, Germany. The powder was then stored in a glass jar with a hermetic lid at room temperature in the dark for advanced assessment. Preceding its addition to the product, the BP powder underwent decontamination using UV laser treatment (TUV 8WT5, Philips, Somerset, NJ, USA) with 8 W of total power, emitting 90% of energy and a 3 min duration time [29].

The BP powder’s moisture, ash, protein, fat, fiber, and carbohydrate (by difference) content was determined according to the established procedures specified in the AOAC [30] standard methodology. The samples were subjected to 3 replicate proximate analyses, and the findings were reported as percentages.

### 2.3. Color Analysis of BP Powder

The color of the BP powder was assessed via a MINOLTA Chroma Meter CR-410 (Konica Minolta, Osaka, Japan) following standardization with a white calibration plate according to the equipment specifications. The specified parameters were L* (lightness/darkness), a* (red/green), and b* (yellow/blue).

### 2.4. Selected Bioactive Compounds Extraction from the Obtained BP Powder

In order to identify specific bioactive compounds in the BP powder obtained, ultrasound-assisted extraction was implemented. A total of 1 g of powdered BP was mixed with 9 mL of a 50% (*v*/*v*) ethanol solution and 1 mL of citric acid (1%), and the mixture was vortexed for 1 min. The extraction was carried out using a sonication bath (MRC. LTD, Holon, Israel) at 40 °C for 50 min at a frequency of 40 kHz. The procedure was performed three times to obtain betalain-rich extracts. Supernatants (pH = 2.19) were then collected after being centrifuged at 5000 rpm for 15 min at 4 °C. In order to characterize the phytochemicals, the supernatant was concentrated at 40 °C under reduced pressure (AVC 2-18, Christ, Shropshire, UK) [31,32].

### 2.5. Extract Characterization

Betalain contents, polyphenols contents, and DPPH radical scavenging activity characterized the BP extract.

#### 2.5.1. Determination of Betalain Pigment Content

The betalain content of BP extracts was determined spectrophotometrically according to the modified protocol used by Šeremet et al. [32] using a UV/Visible Spectrophotometer (Libra S22, Biochrom, UK). The absorbance values were recorded at λ = 538 nm for betacyanins and λ = 480 nm for betaxanthins. The content of betalains was calculated using the Formula (1):

(1) $$\mathrm{Betalains}\;(\mathrm{mg}/g)=\frac{A\;\times \;\mathrm{Vd}\times \;\mathrm{Df}\;\times \;\mathrm{Mw}\;}{m\;\times \;L\;\times \;\xi }$$

where A is the sample absorbance; Vd is the volume of solution; Df is the dilution factor; Mw is molecular weights; L is cuvette pathlength (1 cm); m is the amount of sample; and ξ is the molar extinction coefficients of betacyanins and betaxanthins used to quantify them (Mw = 550 g/mol; ξ = 60,000 L/(mol cm); Mw = 308 g/mol; ξ = 48,000 L/(mol cm) in H_2_O). The total betalain concentration (in mg per g of sample) was calculated by combining the values of betaxanthin and betacyanin.

#### 2.5.2. Determination of Total Phenolic Content

According to Bolea and Vizireanu [33], the Folin–Ciocâlteu method assessed the total phenolic contents. In summary, 7.9 mL of deionized water, 100 µL of the BP extract (1 mg/mL), and 0.5 mL of the Folin–Ciocalteu reagent (0.25 mol/L) were combined. A total of 1.5 mL of a 20% Na_2_CO_3_ solution was added after 10 min, and the mixture was left in the dark for one hour. At λ = 765 nm, the absorbance was finally measured compared to a blank. The total phenolic content was quantified as the gallic acid equivalent per gram of dry weight (mg GAE/g dw) using the gallic acid standard (50–250 mg/L).

#### 2.5.3. Determination of the Antioxidant Activity

The colorimetric technique presented by Shahinuzzaman et al. [34] was slightly modified for the DPPH assay. Briefly, 0.1 mL of BP extract (1 mg/mL) was mixed with 3.9 mL of diluted DPPH or methanol solution (1:10). The mixture was maintained in a reduced brightness environment at room temperature for 90 min. The decrease in the absorbance of the mixture was spectrophotometrically read at λ = 515 nm. The results were reported in µmol Trolox equivalent per gram of dried weight (µmol TE /g DW). The radical scavenging activity was evaluated as the percentage of inhibition, with Trolox serving as the standard (0.125 mg/mL). This was determined using Equation (2):

(2) $$\mathrm{DPPH}\;\mathrm{scavenging}\;\mathrm{activity}\;(\%)=\frac{\mathrm{Abs}\;\mathrm{Control}\;-\;\mathrm{Abs}\;\mathrm{Sample}}{\mathrm{Abs}\;\mathrm{Control}}\times 100$$

where Abs Control is the absorbance value of the DPPH solution only, and Abs Sample is the absorbance value of the DPPH solution mixed with BP extract.

### 2.6. HPLC Investigation of the Betalains from the BP Extract

In order to identify and quantify the betalains pigments from the BP extract, a chromatographic analysis was conducted using the Thermo Finnigan Surveyor HPLC system, controlled by the Xcalibur software system version 2.0.7 (Finnigan Surveyor LC, Thermo Scientific, Waltham, MA, USA).

Solvent A (0.012% aqueous formic acid solution) and solvent B (0.012% formic acid solution and 5% acetonitrile solution) were eluted. The elution gradient was as follows: 0% B (0–0.5 min), 0–90% B (0.5–2 min), 90% B (2–2.5 min), 90–0% B (2.5–2.7 min) and 0% B (2.7–3 min). A volume of 10 μL was utilized for injection, with a flow rate of 1 mL/min. The elution profile was performed with a Synergy 4u Fusion-RP 80A stationary phase column (150 × 4.6 mm, 4 µm). The column temperature was 30 °C. Chromatograms were plotted at 538 nm. The betalains were identified by assessing their retention time and comparing them to established standards and data documented in the literature.

### 2.7. Preparation of Value-Added Meringue Samples

The added-value meringues were obtained from the following ingredients: 27 g egg powder (equivalent to one egg), water (at 40 °C), 50 g powdered sugar, and BP powder (M1—4%, M2—7%, and M3—10%). The procedure described is simple, assuming that the ingredients presented above are mixed with the powder from red BP and added as a bioactive component.

The process of obtaining value-added meringues has the following stages: The egg white powder was dissolved in slightly heated water (40 °C) in a ratio of 1:2 and mixed gently to homogenize. Then, the mixture was mixed with a hand mixer on medium speed for 2–3 min. Once the composition started to foam, the sugar was gradually added in small amounts. After all the sugar is added and the albumen becomes glossy and firm, the composition must be without sugar granules. After the homogeneous composition, the BP powder was added, which was later hydrated with warm water. Mixing was continued in order to make the color uniform. The composition was put in a bag, and the meringues were formed on a baking paper tray. The meringues were introduced in the oven for 60–90 min, at 90 °C, or until the products became firm, but without changing their color to remove them from the baking paper easily. Then, the meringues were left to dry for about 4 h. Finally, the meringues were packed in plastic containers, hermetically sealed, and stored in a dry and cool place.

A control sample (C), which followed the same technology but added no BP powder, was also made for comparison.

### 2.8. Characterization of Phytochemicals, Physicochemical, and Antioxidant Activity of Value-Added Meringue Samples

The physicochemical parameters of meringue samples, such as moisture, protein, fat, ash, total sugar, and caloric values, were assessed using AOAC methodologies [30].

The total betalains, phenolic content, and antioxidant activity of value-added meringues enhanced with BP powder were assessed using the techniques described in Section 2.5 above.

### 2.9. Storage Stability of the Phytochemical Compounds

The meringue samples were kept in light-resistant glass containers at ambient temperature in the dark. They were analyzed for their bioactive contents (betalains and polyphenols) and antioxidant activities, as previously described, during 21 days of storage.

### 2.10. Color Evaluation of Value-Added Meringue Samples

Color attributes of the supplemented meringues containing varying amounts of BP powder were evaluated using a portable colorimeter with the illuminator C (Chroma Meter, model CR-410, Konica Minolta, Osaka, Japan). The results were expressed as lightness (L* where a lower value indicates a darker color, black: L* = 0 and white: L* = 100), a* (indicating the balance between red (>0) and green (<0) color), and b* (the balance between yellow (>0) and blue (<0) color). The CIELAB color attributes (L*, a*, and b*) were acquired in triplicate following equipment calibration against a white plate [35]. The hue angle (arctan(b*/a*) for quadrant I(+a*, +b*), visual color appearance, the Chroma $\left(\sqrt{{\left(a^{*}\right)}^{2}+{\left(b^{*}\right)}^{2}}\right)\;$color intensity, and ΔE $(\sqrt{{\left(L^{*}-L_{0}\right)}^{2}+{\left(a^{*}-a_{0}\right)}^{2}+{\left(b^{*}-b_{0}\right)}^{2}),}$ the total color difference(L_0_, a_0_, and b_0_ represent control values) were also calculated [36].

### 2.11. Textural Parameters of Value-Added Meringue Samples

The texture profile analysis (TPA) approach was used to examine the textural features of the value-added meringue samples using a CT3-1000 Texture Analyzer (Brookfield Ametek, Chandler, AZ, USA). The samples were subjected to double penetration with a metal cylinder with a diameter of 4 mm, up to a depth of 6 mm, and with a speed of 0.5 mm/s. The test speed was set to one millisecond per second, the trigger load to 0.067 N, and the load cell to 9.8 N. The textural parameters firmness, adhesion, cohesiveness, elasticity, and chewiness were calculated using the TexturePro CT V1.5 software (Brookfield Engineering Labs. Inc., Chandler, AZ, USA). Each sample was subjected to three determinations. The samples were maintained at room temperature for two hours before testing.

### 2.12. Sensory Evaluation of Value-Added Meringue Samples

Twenty panelists (aged 25–60 years, 90% women and 10% men) performed a sensory assessment of value-added meringue samples. The sensory analysis involved the selection of twenty untrained panelists, consisting of students and staff members from the Faculty of Food Science and Engineering at the Dunarea de Jos University of Galati. Decision No. 28/19.10.2022, issued by the Dunarea de Jos University Ethics Commission, conducted the sensory evaluation test. They were given information about the study’s overall goal and the protocols for managing personal data. In evaluating the flavor and texture of the value-added meringues, the panelists focused on flavor intensity, the balance of sweetness and earthiness, aroma contribution, color intensity, aftertaste, structure, and firmness, as well as comparing samples with different BP concentrations. The panelists were asked to evaluate the color, appearance, smell, flavor, taste, texture, sound, and aftertaste of value-added meringues using a 9-point hedonic scale (1 = very dislike; 9 = very like). The samples were given three random digit codes and served at room temperature (20–23 °C) and under white light. The order in which the samples were presented was randomized. After each sample evaluation, panelists were advised to rinse their mouths with water [37].

### 2.13. Statistical Analysis

The data was presented as the mean ± standard deviation, and the experiments were conducted in triplicate. ANOVA was performed on the data using Minitab 19 (Minitab Inc., State College, PA, USA). Subsequently, a Tukey’s test was conducted to compare the means at a 5% significance level (*p* < 0.05). Principal components analysis (PCA) was performed on the descriptive sensory data utilizing XLSTAT (Trial Version 2024, Addinsoft, Paris, France).

## 3. Results and Discussions

### 3.1. BP Powder Characterization

The ultrasound-assisted method was used to extract the bioactives from BP powder, and the solvent combination was 50% ethanol acidified with 1% citric acid (ratio 9:1, *v*/*v*). The ethanolic extract of BP showed a high content of biologically active compounds (Table 1). A content of 2.81 ± 0.16 mg betalain/g DW BP and a high concentration of total polyphenols of 51.13 ± 1.14 mg GAE/g DW is noted. High concentrations of bioactive compounds resulted in high antioxidant activity of 48.65 ± 0.63 mM TE/g DW, with a corresponding inhibition of 87.19 ± 1.17%. The L*, a*, and b* color values were 36.61, 30.94, and 4.99 for BP, indicating a reddish color shade.

Table 1 also displays the proximate composition of BP powder. Beetroot peel is a good source of carbohydrates, protein, and fiber, making it a valuable nutritional resource. When comparing the research findings to the published literature, there are several differences in the physicochemical makeup. The results obtained in this study were slightly less than the chemical contents in the beetroot plant, as Neha et al. [38] reported. The study conducted by Shuaibu et al. [39] found differences in the proximate analysis of beetroot peel. It revealed the following composition: moisture content (30.88%), ash content (10.58%), crude fat (3.29%), crude fiber (6.98%), crude protein (4.10%), and carbohydrate (44.17%).

According to Kujala et al. [25], betalain distribution varies across different red beetroot regions. The study found that the exterior sections of red beetroot (cv. Little Ball) had a higher betacyanin content, with the highest levels noticed in the flesh, crown, and peel. Sawicki et al. [40] reported that water/methanol/formic acid mixture (84.95/15/0.05, *v*/*v*/*v*) extracts of BP produced higher total betalain content (17.24 ± 0.06 mg/g DW) than ours. The level of the total betaxanthin content was 4.46 ± 0.03 mg vulgaxanthin I/g DW, and the total betacyanins content was 12.79 ± 0.08 mg betanin/g DW under sonication conditions for 30 s. The antioxidant capacity of the red BP extract was also higher than our results (54.78 ± 2.81 µM TE/g DW). Šeremet et al. [32] reported the phytochemical values of red BP extract using ultrasound-assisted extraction at 200 W and a frequency of 37 kHz for 60 min. The total polyphenol content was 47.99 ± 0.98 mg GAE/g DW, which is lower than our study’s. However, the total betacyanin content (3.84 ± 0.02 mg betanin/g) and total betaxanthin content (6.98 ± 0.06 mg vulgaxanthin I/g) were higher. In addition, the DPPH scavenging activity was 0.048 ± 0.00 mmol Trolox/g DW after the ultrasound-assisted extraction of bioactives from red BP. With the help of ultrasonic extraction (50–60 Hz, 22 °C, 125 W, 30 min), Vulić et al. [41] isolated betalains from the peel and pomace of beetroots using a combination of water and ethanol (1:1) acidified with acetic acid (0.5%). From the BP waste, they identified three main betalains: vulgaxanthin (1.4 to 4.3 mg/g DW), isobetanin (1.2 to 3.1 mg/g DW), and betanin (3.8 to 7.5 mg/g DW). Betanin levels in beetroot pomace extract were considerable (37.22 mg/100g DW). However, the cultivar, various extraction factors (such as the kind of solvent, temperature, and pH), agronomic factors, and the measuring techniques used can all influence the extract’s phytochemical contents.

### 3.2. HPLC Investigation of the Betalains from the BP Extracts

**Figure 1:**
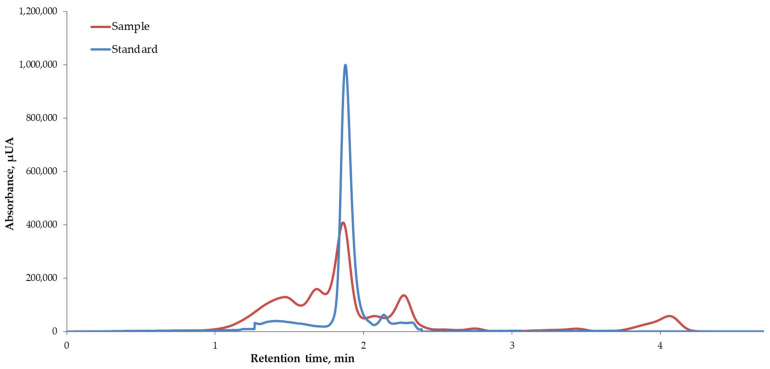
HPLC chromatograms of betalains quantified in the extract of BP.

The chromatographic pattern of the BP extract shows a distinct peak at a retention time of 1.87 ± 0.2 min (Figure 1). Comparing the retention time found in the case of the extract with the chromatogram generated for the betanin standard (1.84 ± 0.01 min), a correlation can be observed between them. It was previously observed in a study conducted by Kujala et al. [42] that the primary betacyanin in red beetroot, betanin, had a predominant distribution in the outer parts of the root. The study focused on the Little Ball cultivar. In contrast, the distribution of betanin decreased in the order of skin, crown, and pulp. According to Rotich et al. [43], betanin is one of the significant beetroot betacyanins, comprising >60% of total pigments, followed by vulgaxanthin I and isobetanin.

The findings are supported by Kathiravan et al. [44] in the case of ready-to-drink beetroot juice samples (*B. vulgaris* L.). The results demonstrated that betanin is the primary compound present in large amounts in the peels and pomace of *B. vulgaris* L. Differences in betalain content and type may be due to varietal diversity, local growth, climatic conditions, and post-harvest conditions [45].

### 3.3. Characterization of the Bioactive Potential of BP-Supplemented Meringues and Storage Stability of the Samples

To highlight the added value of meringues, different percentages of BP powder, 4% (M1), 7% (M2), and 10% (M3), were added, and the phytochemical characterization and antioxidant activity were determined. Additionally, the bioactive compounds’ stability was monitored for 21 days while the meringues were stored at room temperature in hermetically sealed plastic containers, and the results are presented in Table 2.

Table 2 shows that the three variants of meringues with the addition of BP powder showed high concentrations of betalains and polyphenols, which was also reflected in the antioxidant activity values. It can be observed that the BP addition increases significantly (*p* < 0.05) the phytochemicals and antioxidant activity of supplemented meringues when compared to the control on the same day of storage. The presence of phenolic compounds in the control sample was related to the main ingredients used to make the meringues, which were lacking in betalains. Phenolic compounds, which are abundant in plant foods, feature phenolic hydroxyl groups capable of binding to proteins and macromolecules, offering physiological benefits such as antioxidant activity [46]. Bioactive components derived from powdered BP positively impacted the in vitro antioxidant activity of the enhanced meringues. They showed greater antioxidant activity than the control meringues did. Additionally, it is observed that with the increase in the concentration of BP powder, the concentration of biologically active compounds and, implicitly, the antioxidant activity also increases, as expected. Our results comply with other studies. Igual et al. [47] utilized beetroot byproducts to manufacture third-generation value-added snacks, incorporating 25% water content and 10% beetroot byproduct in the corn mixture. The incorporation of beetroot byproduct enhanced the functional value of the snacks, increasing their betalain and phenol content as well as their antioxidant capacity. Sahni and Shere [48] evaluated the acceptability of cookies enriched with beetroot pomace. The use of beetroot pomace enhanced the nutritional and sensory attributes of the enriched biscuit. Cookies using 10% beetroot pomace received the highest sensory approval due to improved taste and flavor, reducing the unattractive darkness typically linked with cookies.

The studies conducted by Mohamed et al. [49] showed that an increased mineral content and antioxidant activity were noted in pasta enriched with beetroot powder (5 g). In accordance with the study of Chhikara et al. [6] regarding vermicelli enhanced with beetroot powder, an improvement in nutritional, physicochemical, and functional quality was observed.

At the same time, however, during the 21 days of storage, the concentrations of betalains and polyphenols decrease significantly (*p* < 0.05) for all the technological variants obtained from the value-added meringues. After storage for 21 days at 24 °C of meringues with the addition of BP powder, a slight decrease in total betalains and total polyphenols and antioxidant activity is observed in all variants of meringues analyzed. Due to processing and storage, there were significant changes in betalains. The stability of betalain during food processing and storage is predominantly influenced by temperature and other significant factors (light, O_2_, pH), as per Sadowska-Bartosz and Bartosz [50]. Betalains and polyphenols degrade during storage due to factors such as heat, light, oxygen exposure, pH fluctuations, and enzymatic activity, leading to color degradation, reduced antioxidant activity, and structural breakdown. Mitigation strategies include controlling temperature, limiting light and oxygen exposure, maintaining optimal pH, and using stabilizing agents [51]. Furthermore, according to Ravichandran et al. [16], applying red beetroot products to thermal treatment results in a reduction of betalains and an enhancement in antioxidant activity. This can be attributed to other antioxidant compounds, such as polyphenols and vitamins, that function together but are influenced differently by temperature and processing [51].

However, the results presented in Table 2 confirm the added value of meringues with the addition of BP powder by increasing the total content of betalains and polyphenols, which leads to a product with high antioxidant activity. These results demonstrate that BP powder can be a natural substitute for chemical colorants.

### 3.4. Physicochemical Characterization of BP-Supplemented Meringue Samples

The value-added meringues were analyzed from a physicochemical point of view, presented in Table 3. The proximate composition of meringues enhanced with BP exhibits substantial differences (*p* < 0.05) compared to the control. Table 3 shows that adding BP powder resulted in a slight decrease in protein content by up to 4% in the M3 sample compared with the control meringues. The moisture content of samples ranged from 7.10 to 9.69%. Incorporating BP in the meringue formulation resulted in a significant reduction in moisture content compared to the control. It has been observed that adding dietary fiber to items in their dry state reduces their moisture content. Similar to our results, Sahni and Shere [48] suggest that physicochemical characteristics (crude fiber, ash, carbohydrates contents) improved by adding beetroot pomace powder to cookies.

The addition of BP powder significantly impacted the ash content in meringues at all levels. Ash content significantly (*p* < 0.05) increased after adding BP powder. Ash content increased from 0.88 to 1.26% for the control and the M3 sample with 10% PP powder, respectively. The observed rise in ash content can be ascribed to the ash content in the vegetable powder.

At the same time, a slight increase in the concentration of carbohydrates is observed with the increase in the percentage of added BP powder. However, the carbohydrate content in M3 rises to 3% relative to the control sample. This increase is also evident in the energy value (Table 3). Additionally, Yadav et al. [52] researched the manufacture of a yogurt-like product fortified with beetroot powder. Their findings indicated that bacterial colonies were maintained, and the protein, carbohydrate, and lipid content increased. Ingle et al. [53] also observed the same trend in the cookie-type product with the addition of beetroot powder.

### 3.5. Color Evaluation of BP-Supplemented Meringue Samples

The results of the color attributes (L*, a*, b*) after obtaining the value-added meringue samples and after 21 days of storage at 24 °C are revealed in Table 4. The addition of BP significantly influenced (*p* < 0.05) all color characteristics of the supplemented meringues. According to the results obtained for the values of a* and b*, all data were placed in the first quadrant (+a*, +b*), suggesting a tendency to be reddish and yellowish, which is characteristic of betalains. Thus, the meringue samples had a reduction in lightness (L*), and lower values indicate a darker color.

According to the results presented in Table 4, value-added meringues are characterized by shades of red (+a*). The intensity of the meringues’ color is directly proportional to the percentage of BP powder added. The results are similar to those of other researchers [54,55].

It is also observed that as the percentage of added powder increases, the brightness of the meringues decreases. Nyam et al. [56] showed that the L* coordinate value reduces considerably when rose seed powder is added. After 21 days of storage, an increase in the importance of the color parameters was observed for all three samples of value-added meringues. Betalains have been utilized as colorants in ice cream, enhancing the product’s acceptance and exhibiting favorable color stability for 180 days when stored at −20 °C [57]. In their study, Rodriguez-Sánchez et al. [58] utilized betaxanthins derived from the fruit of yellow pitaya *(S. pruinosus*) as a pigment for producing jelly gummies. The stability of these pigments in the gummies was attributed to the food matrix, which provided a protective effect through their interactions with proteins and low water activity.

ΔE is the feature of total color change ranging from 47.53 to 68.82 for the meringue samples at the initial moment and 39.05 to 68.11 for the sample at 21 days of storage. Incorporating BP powder significantly influenced (*p* < 0.05) the ΔE values of both control and BP-enriched meringues. Nevertheless, an increase in the BP ratio from 4% to 10% significantly elevates the ΔE. Visual color changes can be classified by total color difference into the following categories: undetectable (0–0.5), moderately noticeable (0.5–1.5), noticeable (1.5–3.0), distinctly visible (3.0–6.0), and significantly different (6.0–12.0) [59]. The Chroma indicating the brightness and saturation of color was highest in the M3 sample and lowest in the control sample. The hue angle value correlated with the received color, signifying the redness of BP powder and BP-enriched meringues since the hue angles were below 10° [60].

These findings indicate that BP powder has a high coloring capacity and can be a natural alternative to chemical dyes.

### 3.6. Textural Properties of BP-Supplemented Meringue Samples

The texture mainly arises from the sensory perception of tactile stimuli resulting from the interaction between a certain body part and the food [61]. The textural parameters—firmness, adhesion, cohesiveness, elasticity, and chewiness of supplemented meringues—were determined, and the results are presented as an average in Table 5. The incorporation of BP markedly influenced (*p* < 0.05) the textural attributes of the BP-supplemented meringues.

Firmness (N) was expressed as the maximum force recorded during the first penetration cycle [61]. Table 5 shows that the minimum value of firmness, 10.44 N, was recorded for the control sample. For the other samples, the firmness values increased simultaneously with the increase in the BP-added powder content. However, analyzing these results from a statistical point of view, it can be noted that these values do not show significant differences (*p* > 0.05). The same can be said about the other texture parameters: adhesion (mJ), expressed as the energy required to overcome the attractive forces between the product and the test device, and cohesiveness (dimensionless size), described as the ability of the product to resist the second deformations, related to the strength during the first deformation [54].

The sample containing 10% BP powder exhibited the maximum elasticity (mm), defined as the degree of deformation recovered following the initial deformation cycle [62]. The maximum chewiness value (mJ), representing the energy necessary to disintegrate the sample during deformation [62], was recorded for the M3 (10%) sample, whereas the minimum value was noted for the control. The texture specifications of hardness, adhesion, cohesiveness, elasticity, and chewiness diminished over the storage period for all examined samples.

Similar values of textural parameters were reported for meringues by Yüceer and Caner [62] and Yüceer and Asik [63]. The results proved that BP powder could obtain meringues with improved nutritional value without significantly influencing the texture. In their study, Salehi et al. [64] found that incorporating 15% dried apple powder into the cake significantly increased overall acceptance. This improvement was achieved by improving the cake’s textural qualities and color.

### 3.7. Sensory Evaluation of BP-Supplemented Meringue Samples

**Figure 2:**
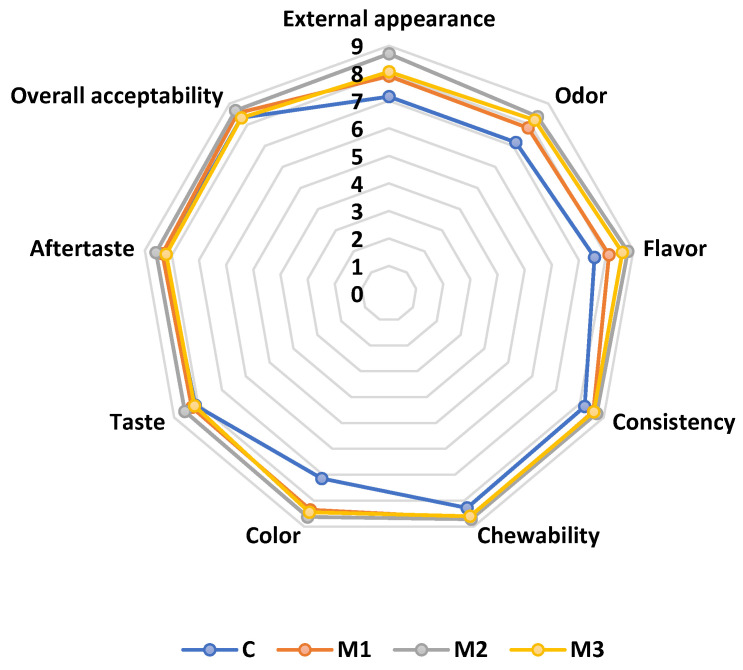
Comparative diagram of the sensory attributes specific to meringues: C-meringues without adding BP powder; M1, M2, and M3—meringues with 4, 7, and 10% powder of BP.

**Figure 3:**
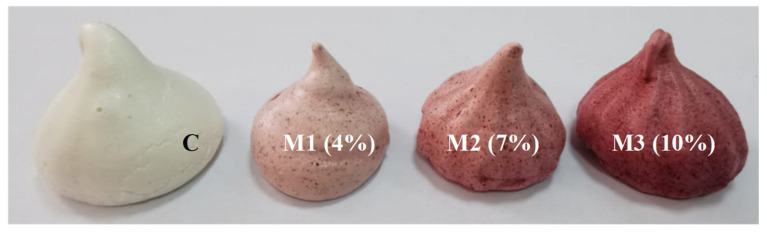
Meringues with different percentages of BP powder: C(control)-meringues without added BP powder, M1, M2, and M3—meringues with 4, 7, and 10% added BP powder.

A nine-point hedonic scale was used to assess the sensory profile of the developed meringues. The sensory analysis followed sensorial attributes such as color, appearance, odor, flavor, taste, consistency, aftertaste, firmness, chewability, and overall acceptability. The resulting meringues presented a consistency specific to the traditional product, a red color specific to beetroot, a sweet, pleasant taste, and a homogeneous texture specific to the conventional product. Analyzing the results of the sensory evaluation of supplemented meringues (Figure 2), it is noted that the variants of meringues with the addition of BP powder were assessed as having a balanced color (Figure 3), pleasant, corresponding to red beet, unlike the control sample which was the least appreciated. All the samples proposed for analysis were positively appreciated by the panelists, who appreciated the value-added meringues as having an easily perceptible beet taste and aroma.

M2, which contains 7% BP powder, is the most appreciated meringue variant. Sample M2 with 7% BP powder obtained the highest scores for most of the analyzed attributes. As the percentage of BP powder increased, the panelists appreciated their taste and aftertaste less.

The panelists appreciated the taste of the meringues as seen by their likeness score for taste. The M1 and M2 meringues’ tastes were comparable to the control. The flavor scores were generally still higher than what was considered acceptable, which was a value of greater than 7.

The tasters noted that the samples differed in color and ranged from light white to reddish-brown, with M3 being the most reddish-brown. The flavor, odor, taste, and aftertaste of the BP powder meringues were well-balanced. The meringues’ crunchy, soft, and non-crumbly consistency was also praised. The panelists evaluated each tasted sample positively and did not detect a strong taste of red BP flavor.

**Figure 4:**
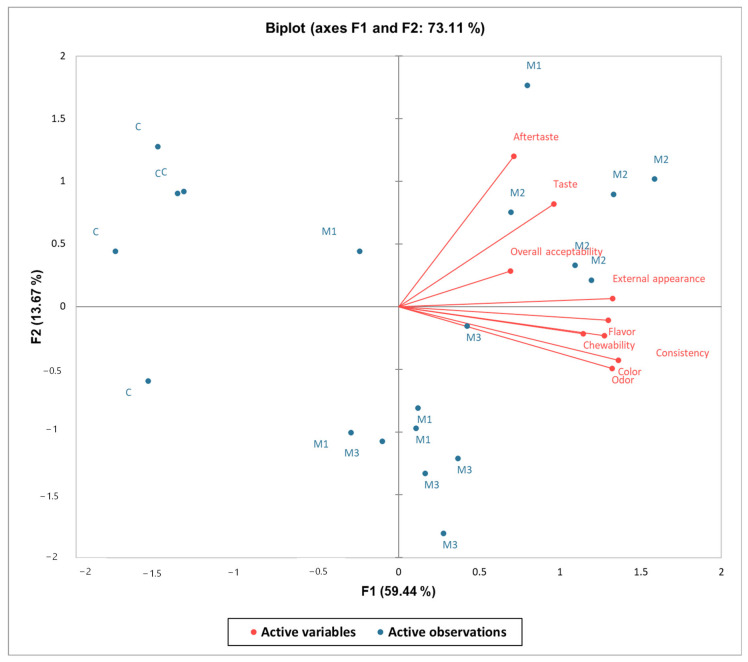
Illustration of correlations among sensory characteristics through Principal Component Analysis (PCA).

The Principal Component Analysis (PCA) (Figure 4) biplot clearly visualizes the variations and relationships between the evaluated sensory attributes and the meringue batches. The attributes found in the upper-right quadrant, namely aftertaste, taste, external appearance, and overall acceptability, were positively positioned in the first axis (F1), signifying a positive correlation. Additionally, color, odor, chewability, consistency, and flavor positively influence axis F1. The placement of these sensory characteristics suggests that M2, located closest to them on the positive side of the biplot, is favorably perceived by consumers regarding taste and overall evaluation. The two axes accounted for 73.11% of the total variation. The C sample was neutral, as all sensory qualities were exclusively connected to the same axis (F1). An analysis of the PCA findings reveals that M2 has emerged as the most prevalent in consumer choices. It was distinguished by a sweet, pleasant taste, a fruity hint, and an appealing appearance.

Results of the sensory evaluation suggest that meringues, in which 7% of BP powder was added, showed positive general acceptability. BP powder could be incorporated up to 7% in the meringue’s recipe without affecting their overall acceptability. Attia et al. [65] assessed the sensory properties of jelly and ice sherbets by employing red beet extract as a colorant in their investigation. The researchers discovered that the concentration of added betalains and the similarity of their properties to those of a synthetic red colorant influenced the overall acceptability of the products.

## 4. Conclusions

The results highlighted that red BP powder is a good source of bioactive compounds with high antioxidant activity. This study highlights BP powder’s potential to enhance meringue’s nutritional and sensory quality, with increased concentrations leading to higher betalains, phenolic content, and antioxidant activity.

The study demonstrates the value of BP powder as a natural pigment and antioxidants in meringues, with sensory evaluations indicating 7% BP powder as the optimal addition for enhanced color, texture, and phytochemical properties. Meringues enriched with BP powder offer improved nutritional value, aligning with consumer preferences for healthier, bioactive-enriched alternatives to traditional meringues. Valorizing red beetroot peels as a source of betalains and polyphenols offers a sustainable alternative to synthetic colorants. Further research is needed to explore their in vivo properties for developing functional food formulations.

## Figures and Tables

**Table 1:** Characterization of the physical, chemical, and functional properties of BP powder.

Parameter	BP Powder
Total betalain, mg/g DW	2.81 ± 0.16
Total phenolic content, mg GAE/g DW	51.13 ± 1.14
Antioxidant activity, µmol TE/g DW	48.65 ± 0.63
Inhibition, %	87.19 ± 1.17
Humidity, %	8.03 ± 0.95
Carbohydrate, %	68.21 ± 0.25
Crude fiber, %	30.89 ± 0.17
Proteins, %	16.90 ± 0.09
Lipids, %	0.49 ± 0.04
Ash, %	6.37 ± 0.52
L*	36.61 ± 0.17
a*	30.94 ± 0.24
b*	4.99 ± 0.06

**Table 2:** Phytochemical characteristics and antioxidant activity of supplemented meringues and stability during 21 days of storage (C-meringues without the addition of BP powder, M1, M2, and M3—meringues with the addition of 4, 7, and 10% (*w*/*w*) BP powder).

Phytochemical Characteristics		Samples			
	Time, Days	C	M1 (4%)	M2 (7%)	M3 (10%)
Total Betalains, mg/100 g DW	0	-	4.10 ± 0.02 ^aA^	6.62 ± 0.05 ^aB^	9.93 ± 0.38 ^aC^
	7	-	3.52 ± 0.04 ^bA^	5.19 ± 0.11 ^bB^	8.38 ± 0.22 ^bC^
	14	-	2,85 ± 0.04 ^cA^	4.58 ± 0.06 ^cB^	7.24 ± 0.11 ^cC^
	21	-	1.60 ± 0.09 ^dA^	3.32 ± 0.18 ^dB^	5.67 ± 0.15 ^dC^
Total Polyphenols, mg GAE/100 g DW	0	38.36 ± 0.29 ^aA^	42.14 ± 1.16 ^aA^	52.80 ± 1.23 ^aB^	65.90 ± 0.68 ^aC^
	7	32.59 ± 0.48 ^bA^	32.26 ± 1.13 ^bA^	40.92 ± 1.44 ^bB^	57.09 ± 0.16 ^bC^
	14	28.87 ± 0.19 ^cA^	30.31 ± 0.85 ^bcA^	36.87 ± 0.29 ^cB^	43.90 ± 0.55 ^cC^
	21	26.01 ± 0.51 ^dA^	28.26 ± 0.41 ^cA^	31.69 ± 0.32 ^dB^	31.18 ± 1.11 ^dB^
Antioxidant activity, µmol TE/100 g DW	0	4.19 ± 0.02 ^aA^	17.15 ± 0.55 ^aB^	27.59 ± 0.74 ^aC^	39.06 ± 0.52 ^aD^
	7	3.90 ± 0.12 ^aA^	14.29 ± 0.98 ^bB^	26.05 ± 1.04 ^aC^	35.01 ± 0.68 ^bD^
	14	2.38 ± 0.18 ^bA^	12.49 ± 0.38 ^bB^	24.65 ± 0.74 ^aC^	29.87 ± 0.87 ^cD^
	21	2.14 ± 0.07 ^bA^	11.94 ± 0.19 ^bB^	22.33 ± 2.35 ^aC^	27.06 ± 0.38 ^dC^

The time variation of compound concentration is highlighted by small letters on the column. Differences in compound concentrations between samples are highlighted by uppercase letters per row. Values that share a lower/uppercase letter are not significantly different (*p* > 0.05).

**Table 3:** Physicochemical characteristics of supplemented meringues (C—meringues without added BP powder, M1, M2, and M3—meringues with the addition of 4, 7, and 10% (*w*/*w*) BP powder).

Physical-Chemical Characteristics	Samples			
	C	M1 (4%)	M2 (7%)	M3 (10%)
Protein, g/100 g	4.81 ± 0.09 ^a^	4.63 ± 0.02 ^a^	4.61 ± 0.03 ^a^	4.60 ± 0.01 ^a^
Carbohydrates, g/100 g	84.62 ± 1.52 ^a^	85.12 ± 3.14 ^a^	86.30 ± 1.09 ^a^	87.04 ± 1.88 ^a^
Humidity, g/100 g	9.69 ± 0.01 ^a^	9.23 ± 0.18 ^a^	7.91 ± 0.36 ^b^	7.10 ± 0.49 ^b^
Ash, g/100 g	0.88 ± 0.02 ^a^	1.02 ± 0.01 ^b^	1.18 ± 0.01 ^c^	1.26 ± 0.02 ^d^
Energetic value, %: Kcal/100 g kJ/100 g	366.66 ± 0.01 ^a^ 1534.10± 0.01 ^a^	367.97 ± 0.02 ^a^ 1539.58 ± 0.02 ^a^	372.73 ± 0.01 ^ab^ 1559.50 ± 0.01 ^ab^	375.72 ± 0.03 ^c^ 1572.01 ± 0.03 ^c^

This means that the same row that does not share a letter differs significantly (*p* < 0.05).

**Table 4:** Colorimetric parameters of the supplemented meringues: C-meringues without adding BP powder, M1, M2, and M3—meringues with adding 4, 7, and 10% (*w*/*w*) BP powder.

Samples	Storage Time (Days)	L*	a*	b*	Chroma	Hue Angle	ΔE
C	0	104.42 ± 0.58 ^aA^	7.72 ± 0.12 ^aD^	1.90 ± 0.04 ^aD^	7.95 ± 0.09 ^aC^	0.24 ± 0.05 ^aB^	-
	21	104.18 ± 0.33 ^aA^	7.43 ± 0.07 ^aD^	1.69 ± 0.02 ^aD^	7.62 ± 0.12 ^aC^	0.22 ± 0.06 ^aB^	-
M1 (4%)	0	60.86 ± 0.77 ^bB^	26.70 ± 0.49 ^bC^	2.87 ± 0.05 ^bC^	26.85 ± 0.14 ^bB^	0.11 ± 0.03 ^aA^	47.53 ± 0.24 ^aC^
	21	70.52 ± 0.19 ^aB^	27.11 ± 0.72 ^aC^	3.80 ± 0.12 ^aC^	27.38 ± 0.19 ^aB^	0.14 ± 0.02 ^aA^	39.05 ± 0.18 ^bC^
M2 (7%)	0	50.95 ± 1.01 ^bC^	32.24 ± 0.42 ^aA^	5.37 ± 0.54 ^bA^	32.68 ± 0.20 ^bA^	0.17 ± 0.04 ^aA^	58.93 ± 0.27 ^aB^
	21	59.67 ± 0.60 ^aC^	32.91 ± 0.23 ^aA^	6.29 ± 0.09 ^aA^	33.51 ± 0.22 ^aA^	0.19 ± 0.07 ^aA^	51.49 ± 0.28 ^bB^
M3 (10%)	0	39.67 ± 0.60 ^aD^	30.91 ± 0.23 ^bB^	4.29 ± 0.09 ^bB^	31.21 ± 0.24 ^bA^	0.14 ± 0.04 ^bA^	68.82 ± 0.35 ^aA^
	21	39.89 ± 0.78 ^aD^	31.68 ± 1.07 ^aB^	5.61 ± 0.09 ^aB^	32.17 ± 0.25 ^aA^	0.18 ± 0.05 ^aA^	68.11 ± 0.34 ^aA^

Small letters on the column highlight color variation over time. Capital letters in a column highlight the color differences between the samples. Values that share a lower/uppercase letter are not significantly different (*p* > 0.05).

**Table 5:** Textural parameters of the supplemented meringues: C-meringues without adding BP powder, M1, M2, and M3—meringues with adding 4, 7, and 10% (*w*/*w*) BP powder.

Parameter	Storage Time (Days)	C	M1 (4%)	M2 (7%)	M3 (10%)
Firmness, N	0	10.44 ± 0.77 ^aA^	10.50 ± 0.99 ^aA^	10.57 ± 0.90 ^aA^	10.92 ± 0.14 ^aA^
	21	10.23 ± 0.67 ^aB^	10.32 ± 0.79 ^aB^	10.28 ± 0.81 ^aB^	10.61 ± 0.19 ^aB^
Adhesion, mJ	0	0.30 ± 0.03 ^aA^	0.28 ± 0.01 ^aA^	0.28 ± 0.01 ^aA^	0.31 ± 0.02 ^aA^
	21	0.19 ± 0.02 ^aA^	0.15 ± 0.01 ^aA^	0.16 ± 0.01 ^aA^	0.20 ± 0.02 ^aA^
Cohesiveness, -	0	0.05 ± 0.01 ^aA^	0.06 ± 0.01 ^aA^	0.06 ± 0.01 ^aA^	0.06 ± 0.01 ^aA^
	21	0.02 ± 0.01 ^aA^	0.03 ± 0.01 ^aA^	0.03 ± 0.01 ^aA^	0.02 ± 0.01 ^aA^
Elasticity, mm	0	2.12 ± 0.15 ^abA^	2.18 ± 0.03 ^abA^	2.06 ± 0.11 ^aA^	2.30 ± 0.07 ^bA^
	21	1.93 ± 0.13 ^abA^	1.88 ± 0.07 ^abA^	1.71 ± 0.10 ^aB^	2.11 ± 0.06 ^bB^
Chewiness, mJ	0	1.72 ± 0.10 ^aA^	1.83 ± 0.01 ^abA^	1.83 ± 0.17 ^abA^	2.00 ± 0.16 ^bA^
	21	1.51 ± 0.11 ^aB^	1.62 ± 0.01 ^abB^	1.63 ± 0.09 ^abB^	1.79 ± 0.15 ^bB^

Lowercase letters per row highlighted differences between the analyzed samples. Capital letters in the column highlight textural parameters’ variation over time. Mean values that share a lower/uppercase letter are not significantly different (*p* > 0.05).

## Data Availability

The original contributions presented in this study are included in the article. Further inquiries can be directed to the corresponding author.
